# Supplementary material for: Extreme magnetoresistance at high-mobility oxide heterointerfaces with dynamic defect tunability
Source: Nat Commun. 2024 May 18;15:4249. doi: 10.1038/s41467-024-48398-8 (PMC11102559; doi:10.1038/s41467-024-48398-8)
Supplement: Supplementary file 1 — Supplementary Information [file 41467_2024_48398_MOESM1_ESM.pdf]

Extreme Magnetoresistance at High-Mobility Oxide Heterointerfaces with Dynamic Defect Tunability

- Supplementary information

D. V. Christensen[1†], T. S. Steegemans[1], T. D. Pomar[1],
Y. Z. Chen[1,2], A. Smith[1], V. N. Strocov[3], B. Kalisky[4], and N. Pryds[1]

Affiliations:
[1] *Department of Energy Conversion and Storage, Technical University of Denmark, DK-2800 Kongens Lyngby, Denmark.*
[2] *Beijing National Laboratory for Condensed Matter Physics and Institute of Physics, Chinese Academy of Sciences, Beijing 100190, China.*
[3] *Swiss Light Source, Paul Scherrer Institute, 5232 Villigen-PSI, Switzerland.*
[4] *Department of Physics and Institute of Nanotechnology and Advanced Materials, Bar-Ilan University, Ramat-Gan 5290002, Israel.*

[†] Email: dechr@dtu.dk

List of Content:

Supplementary Section 1: Magnetic field dependence of the magnetoresistance 2
Supplementary Section 2: Reproduction of magnetoresistive behavior 3
Supplementary Section 3: Arrhenius plot 4
Supplementary Section 4: Field/temperature phase diagrams 5
Supplementary Section 5: Room temperature aging 6
Supplementary Section 6: Annealing 7
Supplementary Section 7: Large-area scanning SQUID microscopy 9
Supplementary Section 8: Magnetoresistive scaling 10
Supplementary Section 9: Kohler scaling 11
Supplementary Section 10: Origin of the extreme magnetoresistance and its tunability 12
Supplementary Section 11: Geometric dependence of the magnetoresistance 16
Supplementary Section 12: Field-dependence of the conductivity tensor elements 17
Supplementary Section 13: Anisotropy 20
Supplementary Section 14: References 21

Supplementary Section 1: Magnetic field dependence of the magnetoresistance

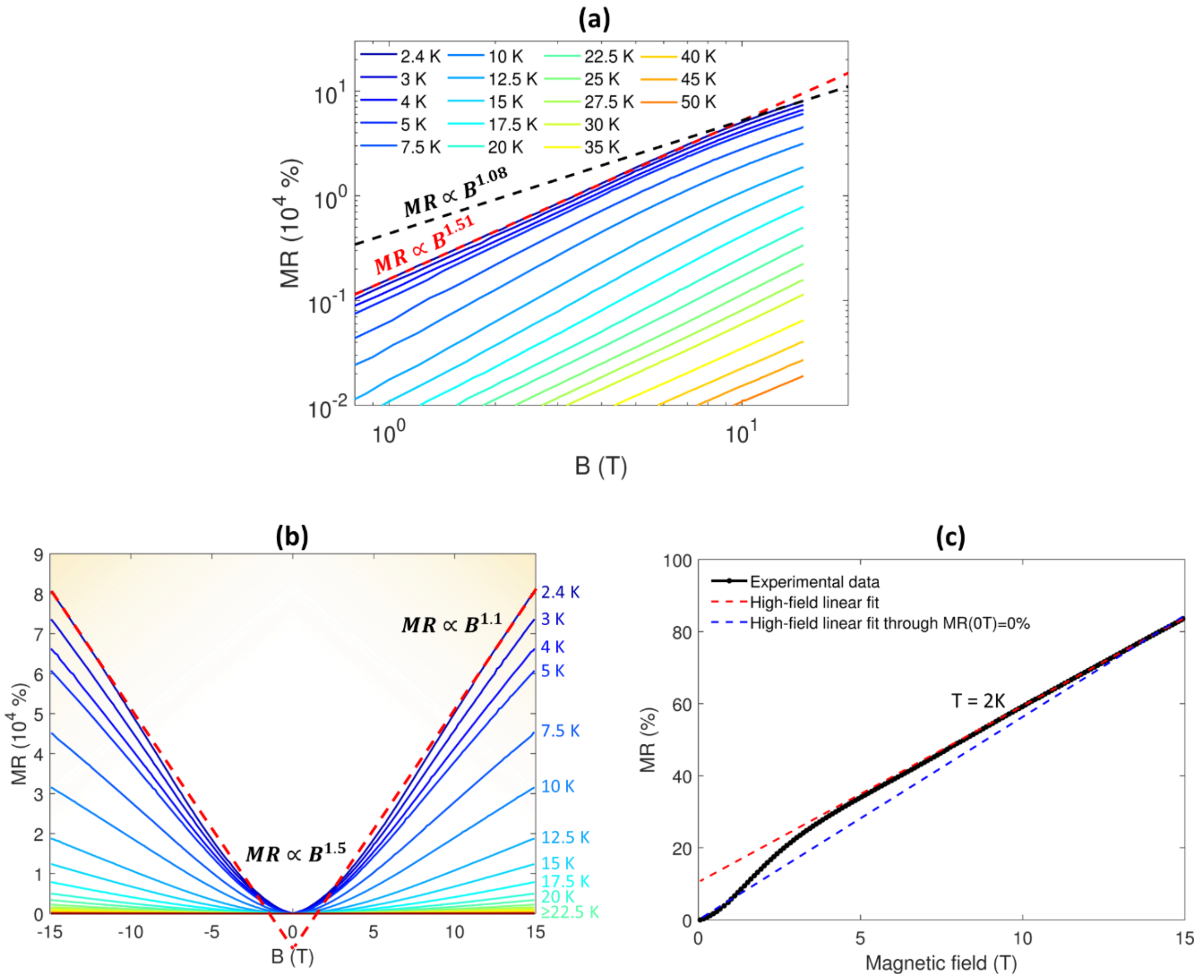

Figure S1: (a) *Double logarithmic plot of the magnetoresistance (MR) as a function of magnetic field (B) for temperatures ranging from 2.4 to 50 K for sample 6b, which is the same sample displayed in Figure 1 and 2 in the main text. At low temperatures, the magnetoresistance scales as* $MR \propto B^{1.51}$ *for low to moderate magnetic fields and as* $MR \propto B^{1.08}$ *for high magnetic fields. Magnetoresistance at a linear scale with the high-field dependence extrapolated to B = 0 T for **(b)** sample 6b and **(c)** another sample.*

Supplementary Section 2: Reproduction of magnetoresistive behavior

***Figure S2**: Temperature-dependent magnetoresistance (MR) as a function of magnetic field (left column) and the field-dependent sheet resistance as a function of temperature (right column) displayed for five different γ-Al_2O_3/$SrTiO_3$ heterostructures.*

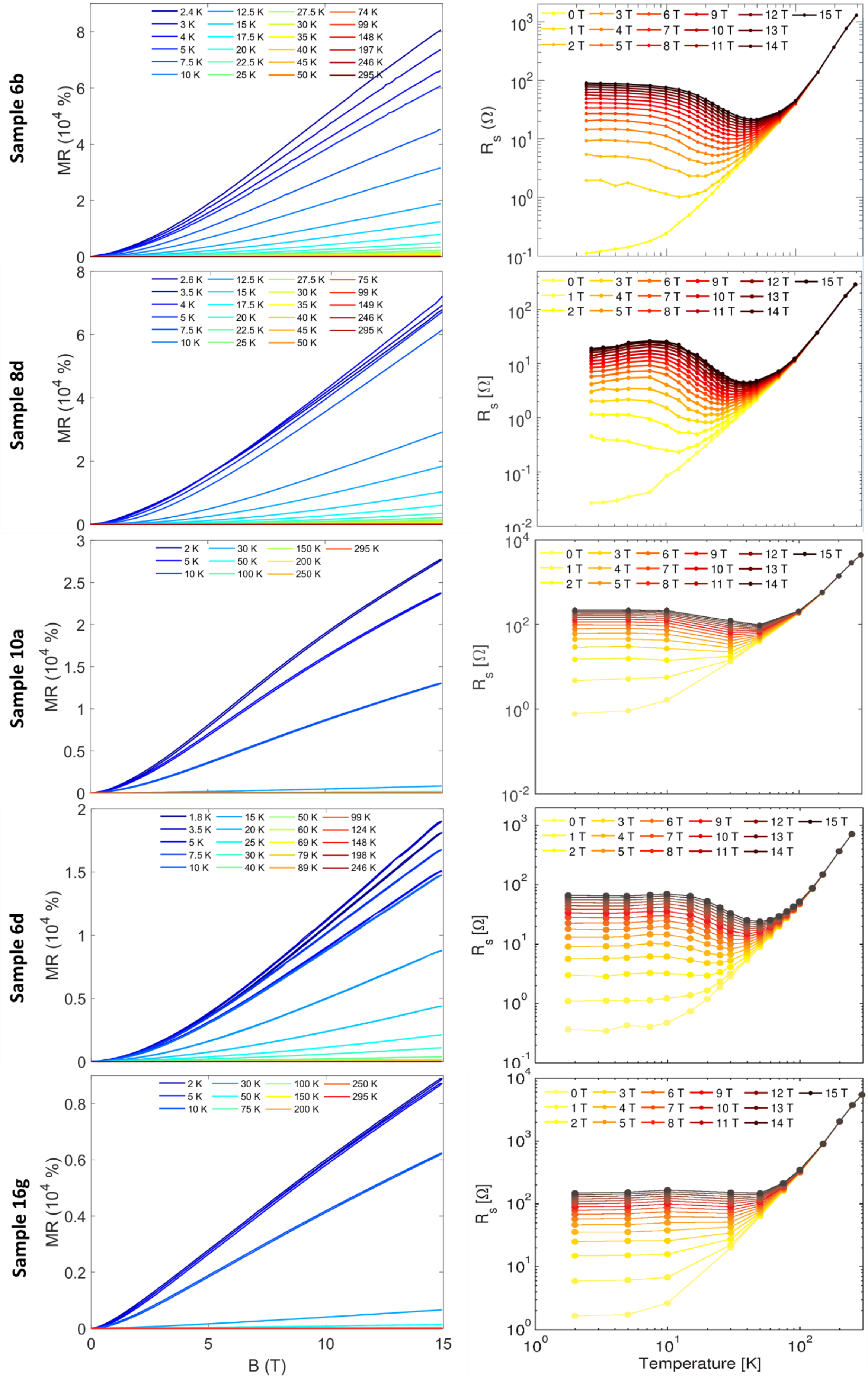

Supplementary Section 3: Arrhenius plot

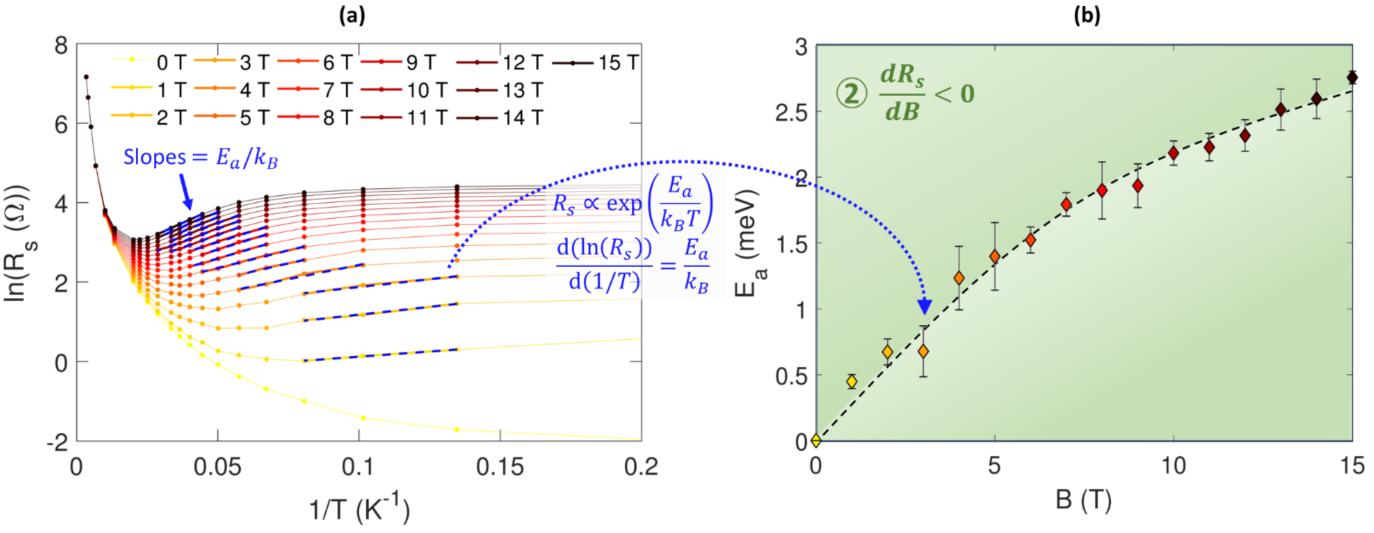

Figure S3**:** ***(a)*** *Arrhenius plot showing how the logarithm of the sheet resistance varies with the inverse temperature assuming a thermally activated behavior according to* $R_s \propto exp\left(\frac{E_a}{k_B T}\right)$ *(1). In region 2 described in the main text, the resistance increases when lowering the temperature. This region is approximately linear on the Arrhenius plot and fitted using linear regression as displayed with the dashed lines.* ***(b)*** *The activation energy extracted from the Arrhenius plot and illustrated as a function of the magnetic field suggests that the decrease in sheet resistance when heating the sample within region 2 is a thermally activated process with an associated energy on the order of a few meV. The error bars describe the standard deviation of the linear fit in the Arrhenius plot. The black dashed line is a guide to the eye.*

Supplementary Section 4: Field/temperature phase diagrams

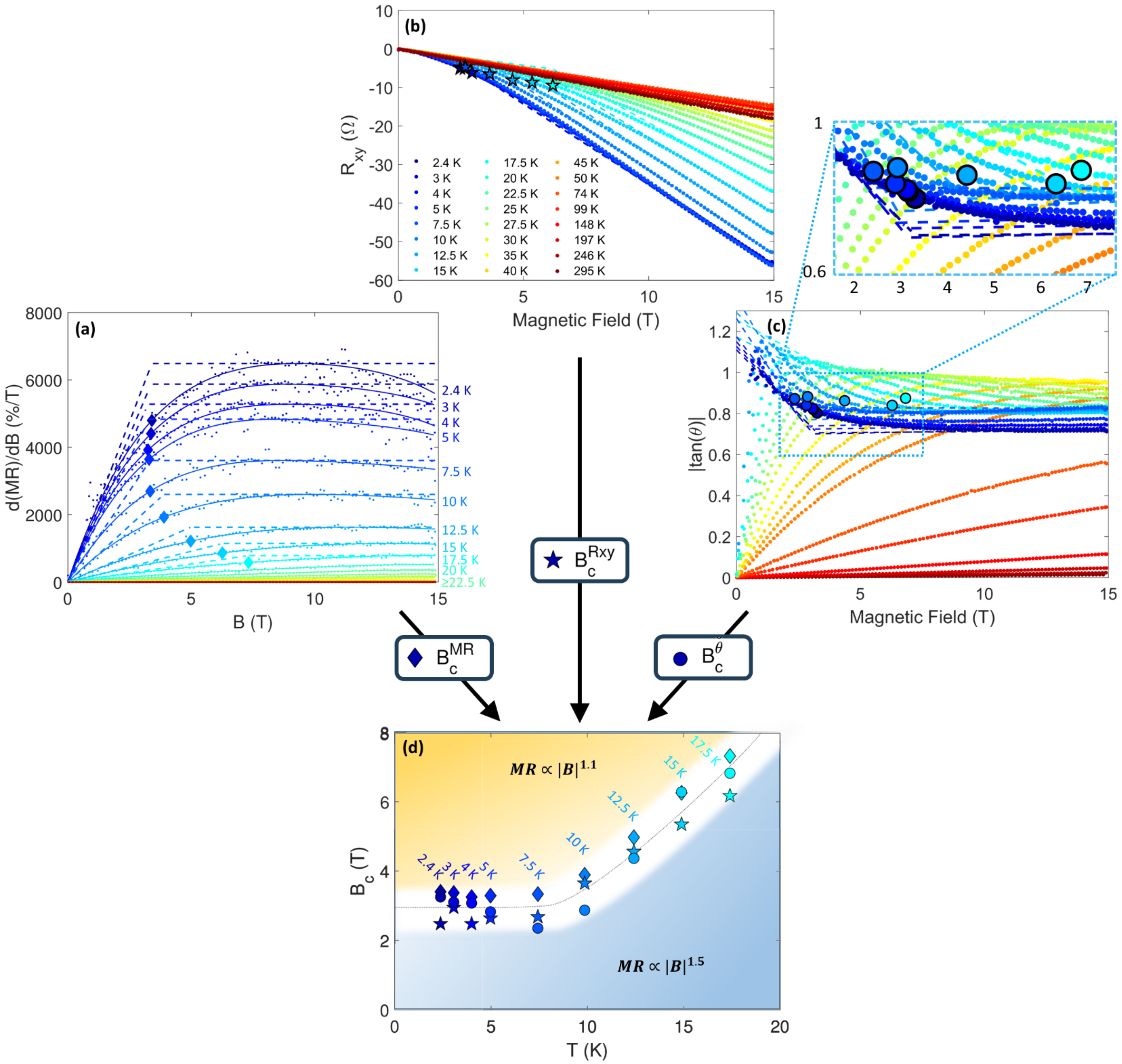

***Figure S4**: **(a)** Derivative of the magnetoresistance with respect to the magnetic field, d(MR)/dB, as a function of the magnetic field where linear fits at low and high magnetic fields are used to define a characteristic magnetic field denoting the onset of linear magnetoresistance. **(b)** Transverse resistance as a function of magnetic field with linear fits at low and high magnetic fields used to define the inflection point. **(c)** Tangent to the Hall angle displayed as a function of the magnetic fields with linear fits again used to define the onset of a field-independent Hall angle. **(d)** Phase diagram showing how the three characteristic fields depend on temperature.*

Supplementary Section 5: Room temperature aging

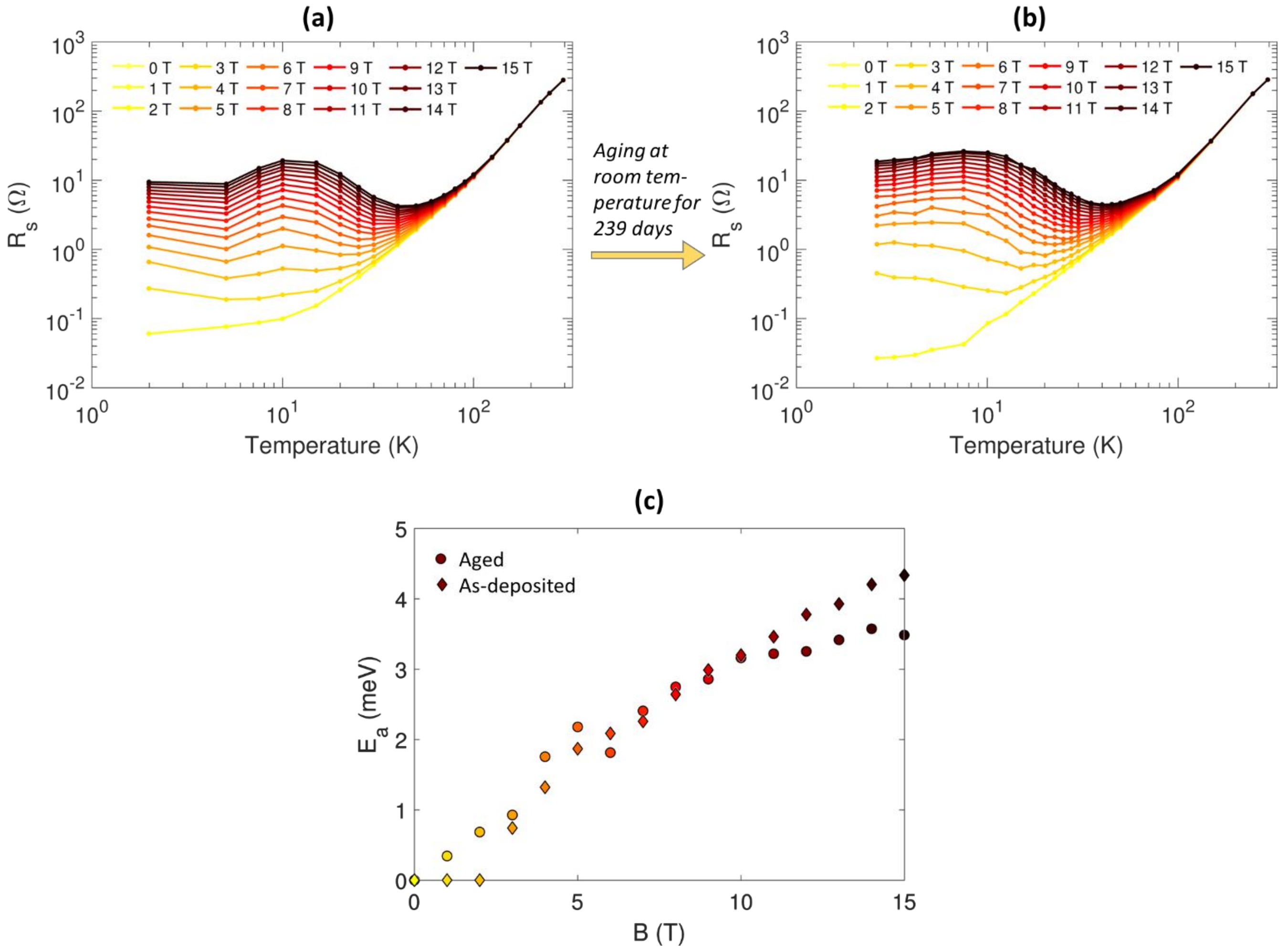

Figure S5 (top figures): *Sheet resistance (R_s) as a function of temperature displayed for various temperatures* ***(a)*** *prior and* ***(b)*** *after room temperature storage for 239 days.* ***(c)*** *The associated activation barriers (E_a) before and after annealing extracted as in Figure S3.*

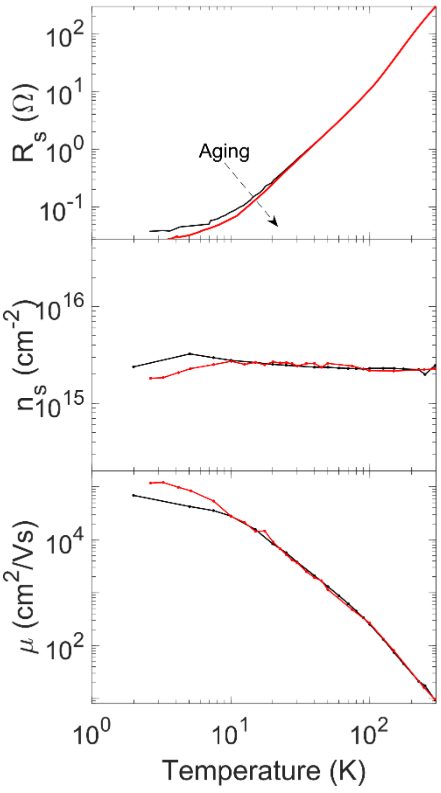

Figure S6 (right figures): *Temperature dependent* ***(a)*** *sheet resistance (R_s),* ***(b)*** *sheet carrier density (n_s) and* ***(c)*** *zero-field electron mobility (μ) prior and after room temperature storage for 239 days. The sheet carrier density and electron mobility are extracted from the linear Hall slope around zero magnetic field. Similar trends are seen in other γ-Al_2O_3/$SrTiO_3$ heterostructures as shown elsewhere (2).*

Supplementary Section 6: Annealing

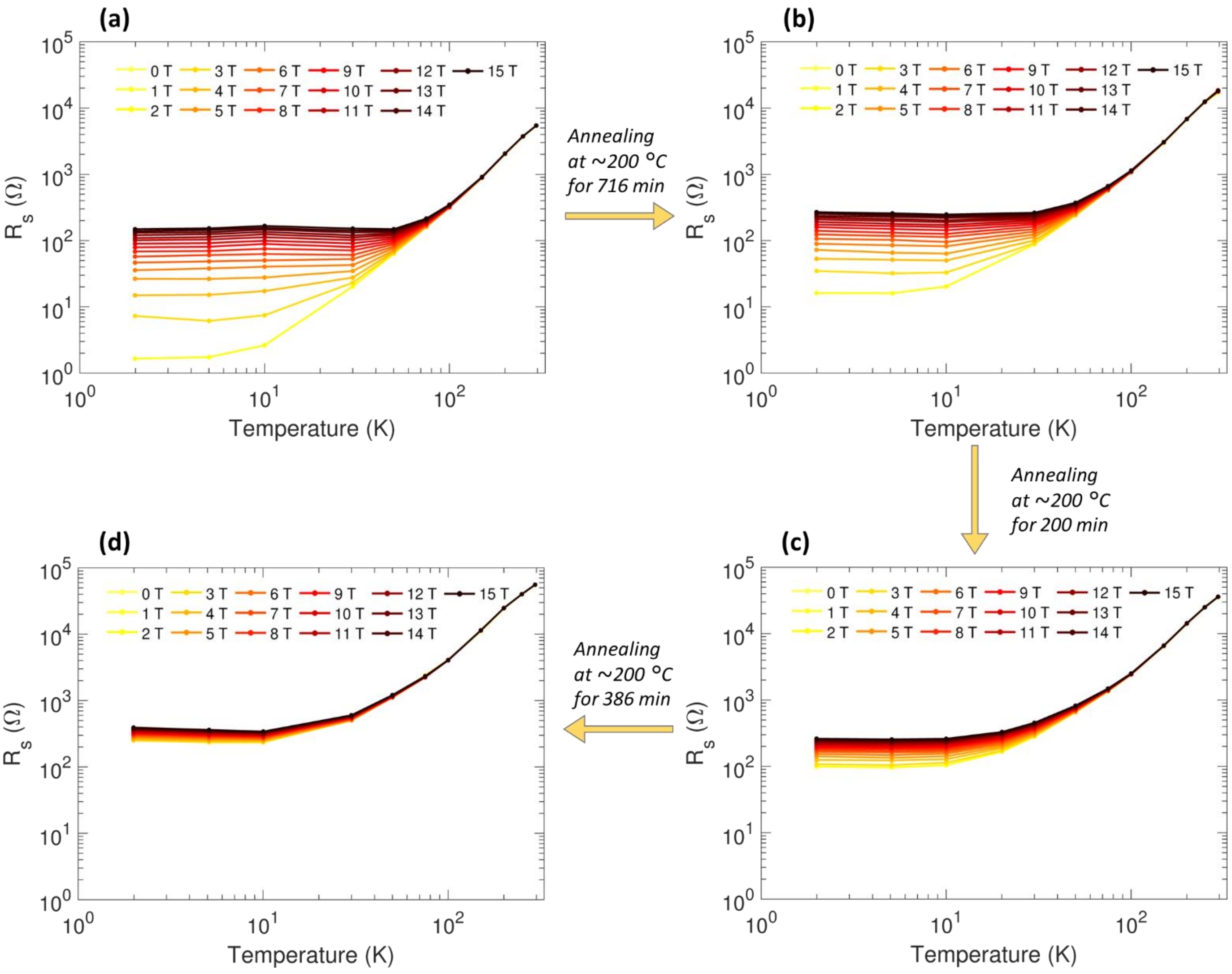

Figure S7: *Sheet resistance (R_s) as a function of temperature displayed for various temperatures* ***(a)*** *prior to annealing and* ***(b-d)*** *after three consecutive annealing steps at 200 °C in oxygen.*

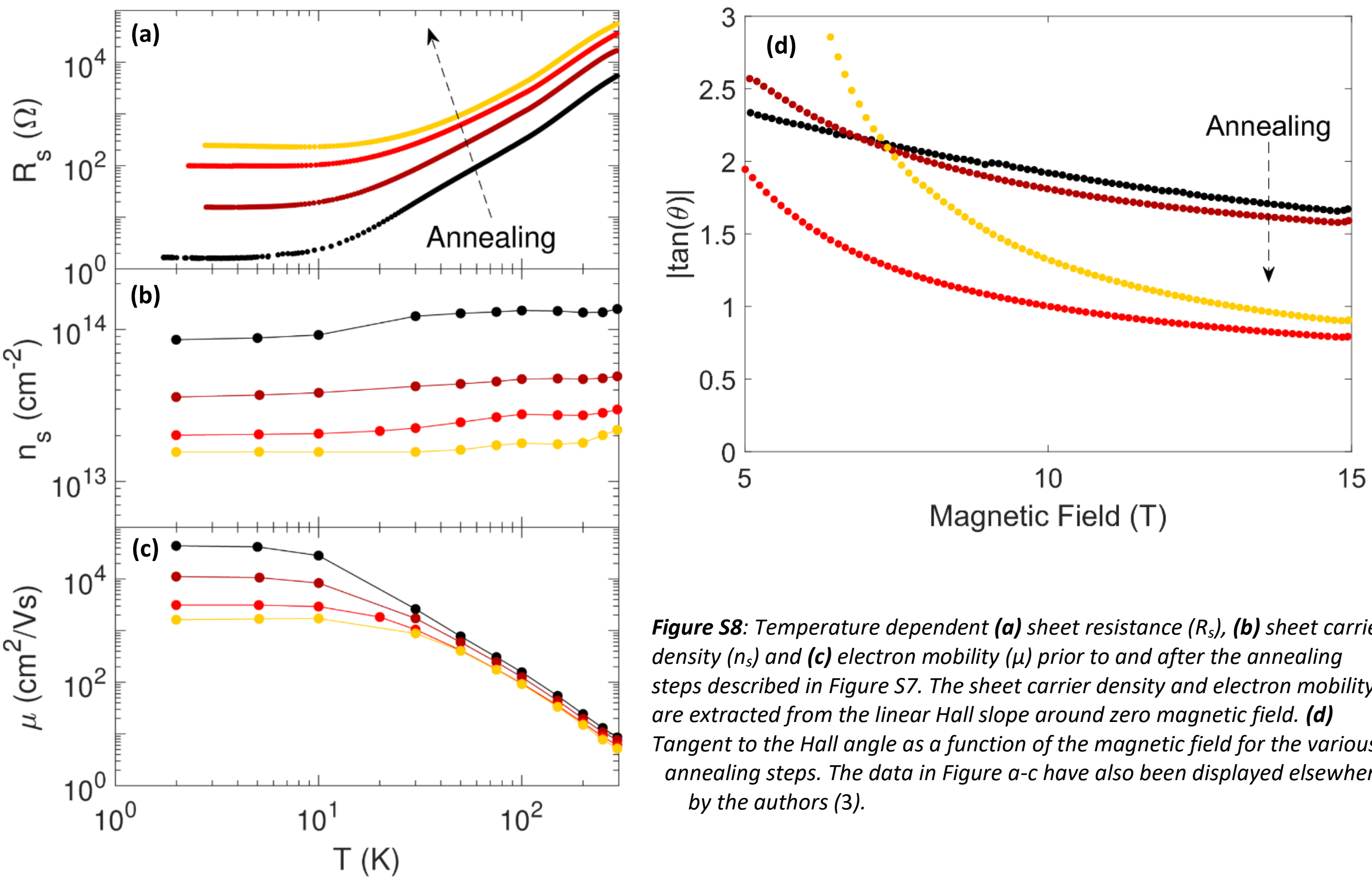

Figure S8: *Temperature dependent* ***(a)*** *sheet resistance (R_s),* ***(b)*** *sheet carrier density (n_s) and* ***(c)*** *electron mobility (μ) prior to and after the annealing steps described in Figure S7. The sheet carrier density and electron mobility are extracted from the linear Hall slope around zero magnetic field.* ***(d)*** *Tangent to the Hall angle as a function of the magnetic field for the various annealing steps. The data in Figure a-c have also been displayed elsewhere by the authors (3).*

Supplementary Section 7: Large-area scanning SQUID microscopy

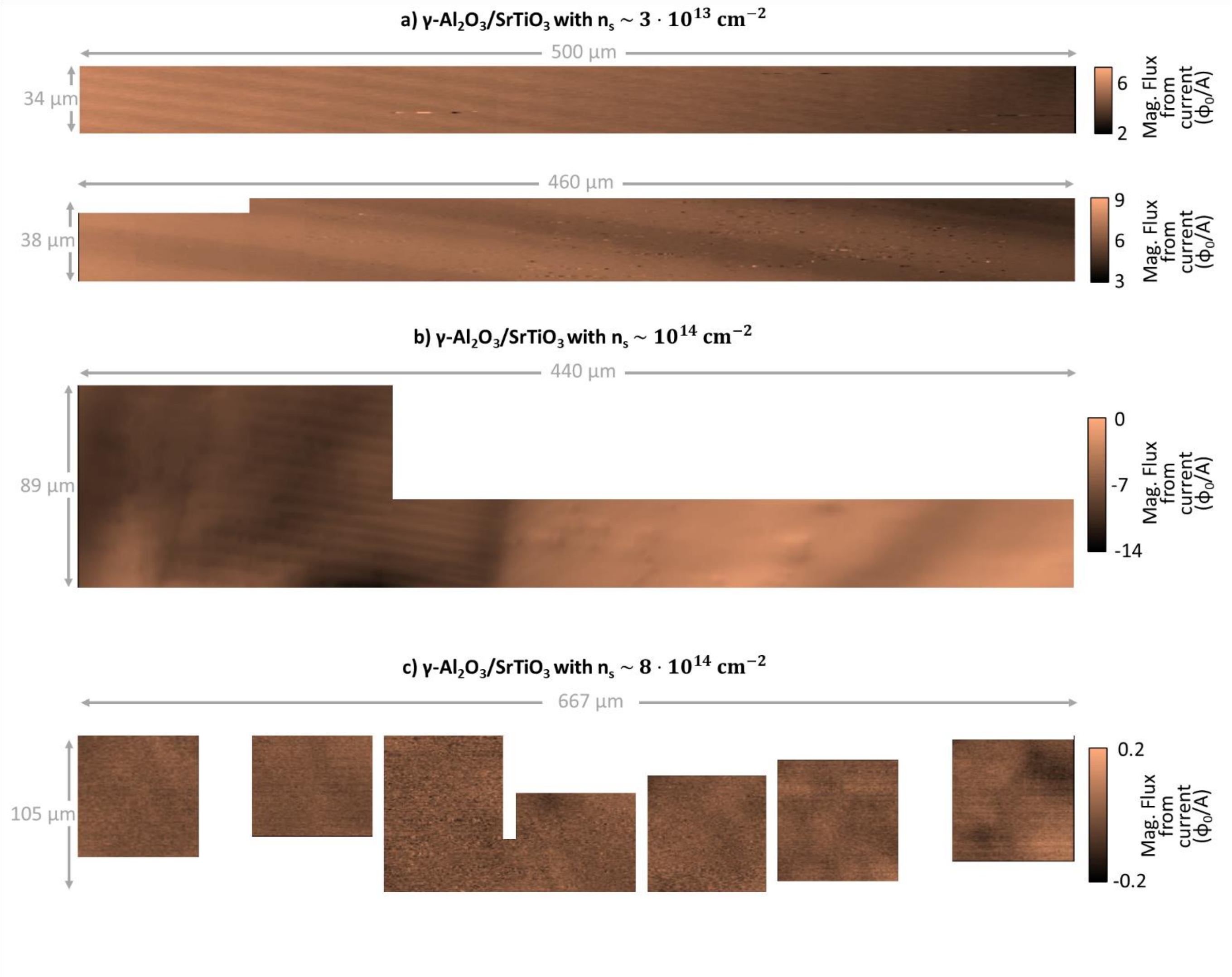

Figure S9: The magnetic flux from an alternating current detected using a scanning superconducting quantum interference device (SQUID). (a-c) Several scanning SQUID scans have been merged to produce a large-area view of the local current inhomogeneities in three γ-Al_2O_3/$SrTiO_3$ heterostructures with varying sheet carrier densities (n_s). In panel c, we removed large-scale variations from the data by a high pass filter to get a better view of the local changes in the magnetic field distribution. The large-scale signals indicate the overall direction of the current flow.

Supplementary Section 8: Magnetoresistive scaling

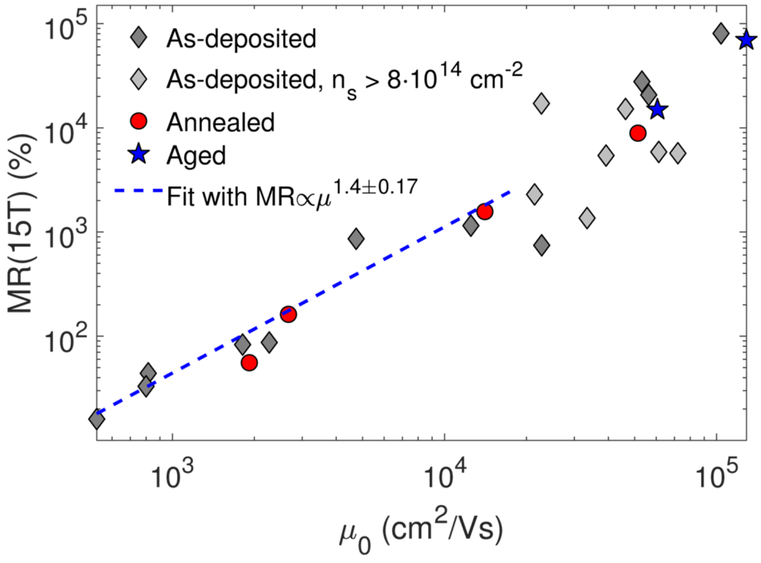

***Figure S10**: Magnetoresistance (MR) at 15 T as a function of the zero-field mobility (μ_0) presented for a range of samples with variations in the deposition parameters or post-processing through annealing and aging as described in the main text.*
Fitting the magnetoresistance as a function of the mobility reveals that $MR \propto \mu_0^{1.4\pm0.17}$ for the compiled data with $\mu_0 < 20{,}000$ cm²/Vs. For data with higher mobility, the trend remains positive but with an imprecise scaling relationship as the data is more scattered in this region. The scattered points originate from sample-to-sample variations as well as from the inclusion of data with $n_s > 8 \cdot 10^{14}$ cm⁻² where bulk conductivity is likely to start emerging.

Supplementary Section 9: Kohler scaling

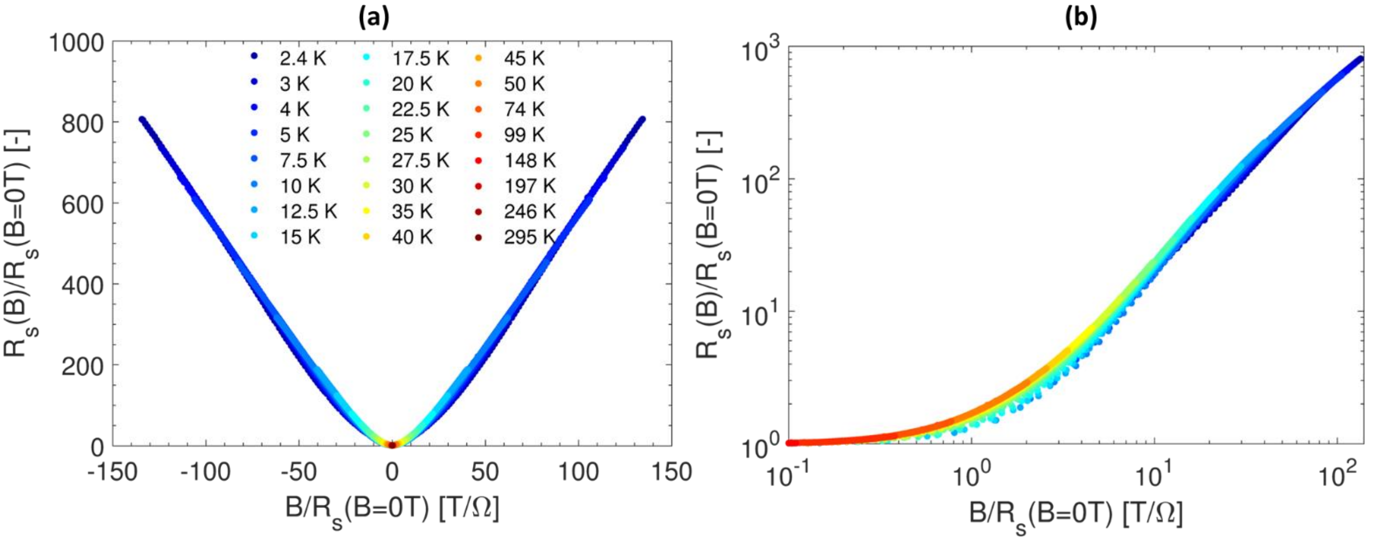

Figure S11: *Kohler scaling on a **(a)** linear plot and **(b)** double-logarithmic plot showing an overall reasonable collapse of the field dependent sheet resistance (R_s) normalized with the zero-field sheet resistance when these are plotted against the ratio B/R_s. Despite collapsing the curves to a fair extent, the scaling is not perfect. In particular, deviations of Kohler's rule were previously found at temperatures below 40 K for γ-Al_2O_3/$SrTiO_3$, which was attributed to interactions between itinerant electrons and a magnetic order. This interaction was inferred to cause a negative magnetoresistance contribution to be added to an overall positive magnetoresistance of γ-Al_2O_3/$SrTiO_3$ (4).*

Supplementary Section 10: Origin of the extreme magnetoresistance and its tunability

We here turn our attention to the origin of the high magnetoresistance and its linear behavior at high magnetic fields. The most common mechanism for archetypical XMR materials is charge compensation (*5*). Despite that the universal triangular phase diagram is also observed for γ-Al_2O_3/$SrTiO_3$, the linear rather than quadratic magnetoresistance combined with a band structure comprising only *n*-type carriers exclude this mechanism. The ARPES data further show that the unsaturated MR of γ-Al_2O_3/$SrTiO_3$ also does not come from an open Fermi surface (*6*) and that heavy bands are present in γ-Al_2O_3/$SrTiO_3$ rather than the steep bands observed to promote the mobility and magnetoresistance in many XMR semimetals.

The linear magnetoresistance is unlikely to be resulting from transport in the extreme quantum limit as it would require a carrier density below $(eB/\hbar)^{3/2} = 3 \cdot 10^{17}\ \mathrm{cm}^{-3}$ (*7*, *8*). This carrier density is similar to the high-mobility $SrTiO_3$ thin films grown with very low doping of $SrTiO_3$ by molecular beam epitaxy (*9*), but much lower than that obtained in γ-Al_2O_3/$SrTiO_3$ and generally other $SrTiO_3$-based heterointerfaces grown by pulsed laser deposition (*10*, *11*).

In contrast, the linear magnetoresistance is likely to arise from inhomogeneities in the conductivity from either a strongly disordered material in the classical transport regime (*12*, *13*) or a weakly disordered material in the semiclassical regime (*14*). The geometric aspect of the magnetoresistance is consistent with Figure S14, which probes the geometric contribution to the magnetoresistance by comparing magnetotransport with a van der Pauw and linear contact configuration. The degree of disorder can be assessed through the thermal energy required to relax the disorder-induced high-resistive state (region 2 of Figure 1 in the main text). This energy (a few meV, see Figure 2b) is much smaller than the measured Fermi energy level (several tens of meV, see Figure 4e), which classifies the γ-Al_2O_3/$SrTiO_3$ heterostructures with high MR as a weakly disordered medium. The claim of weak disorder is further supported by the high mobility and the slowly varying magnetic stray field for the heterostructures with high linear magnetoresistance. From the magnetoresistive scaling, the linear magnetoresistance is observed for values of $\mu_0 B > 10$ for the sample presented in Figure 4g, which places the transport in the semiclassical regime. Therefore, the magnetoresistance in γ-Al_2O_3/$SrTiO_3$ appears to be consistent with the guiding-center model in the semi-classical transport regime with weak disorder. The guiding center model applies to a conducting medium with a 3D or quasi-2D character with a slowly varying disorder potential compared to the cyclotron radius. In addition, a key experimental hallmark is a field-independent Hall angle. As we argue below, these assumptions are all fulfilled in high-mobility γ-Al_2O_3/$SrTiO_3$.

Spatial extent of the electron gas:

The large, linear magnetoresistance emerges at high sheet carrier densities on the order of $n_s = 5 \cdot 10^{14}$ cm^{-2} and can be further enhanced by aging. The high sheet carrier density, the transition in the local current distribution observed by scanning SQUID as well as the ARPES data point towards an extended depth distribution of the itinerant charges. This is supported by the predominant population of heavy 3D bands and the enhancement of the magnetoresistance through aging, which both boost the mobility and are predicted to expand the depth distribution (*10*, *15*). The depth distribution of γ-Al_2O_3/$SrTiO_3$ samples with similar mobility and carrier density has been assessed by angle-dependent Shubnikov-de Haas oscillations (*16*), angle-dependent X-ray photoemission spectroscopy (*16*), hard x-ray photoemission spectroscopy (*17*) and infrared ellipsometry (*18*), which point towards the majority of electrons residing within the first 1-10 nm of the γ-Al_2O_3/$SrTiO_3$ interface. This indicates that a significant fraction of the carriers resides in an interface-near region of $SrTiO_3$ with confinement formed by oxygen vacancies stabilized at the γ-Al_2O_3/$SrTiO_3$ interface, whereas the remaining electrons distribute deeper into $SrTiO_3$. This picture is consistent with numerical simulations on γ-Al_2O_3/$SrTiO_3$ (*2*, *10*).

Slowly varying disorder potential:

The characteristic length (ξ) of the disordered potential variations can be estimated by $\xi \gg r_c = \frac{\hbar k_F}{e B_c^{MR}}$ where r_c, k_F and B_c^{MR} denote the cyclotron radius, Fermi momentum and crossover magnetic field to the linear magnetoresistance, respectively (*14*). For Fermi momenta of the heavy bands along the heavy and light direction, k_F are 0.34 Å^{-1} and 0.069 Å^{-1} (Figure 4e) yielding cyclotron radii of 750 and 150 nm at $B_c^{MR}\sim$3 T at 2 K (Figure 2c), respectively. Hence, the characteristic disorder length is expected to be on the scale of several hundreds of nanometers to micrometers. In contrast to XMR materials with steep bands, the heavy bands and large carrier density of γ-Al_2O_3/$SrTiO_3$ also translate into a large Fermi momentum, which sets the lower bound for the characteristic disorder length. Combined with a

very high dielectric constant, the characteristic disorder length in γ-Al_2O_3/$SrTiO_3$ is an order of magnitude larger than in the 3D Dirac metals discussed in Ref. (*14*). The large, linear magnetoresistance emerges at high sheet carrier densities where the magnetic field from the local current distribution has transitioned from a stripe behavior to slowly varying modulations with a characteristic length of a few tens of micrometers (Figure 4a). We stress that the Oersted field may be broadened by the distance between the pick-up loop and the spatially extended current, and that the actual modulation of the current may have finer details not resolvable here. Irrespective of this, the scanning SQUID images justify the assumption of a slowly varying disorder potential. This is further supported by the high dielectric constant of $SrTiO_3$ exceeding 10,000 (*19*), which favors a slowly varying disorder potential by screening scattering sites and inhomogeneities. The scanning SQUID images (Figure 4a) also reveal that the striped current modulations and magnetic order (*4*) along the tetragonal domain walls are not a likely origin of disorder and linear magnetoresistance.

Saturating Hall angle:
As depicted in Figure 2c, $\tan(\theta_H)$ saturates above a characteristic crossover magnetic field, which coincides with the emergence of linear magnetoresistance. In this case,

$$R_s t = \rho_{xx}(B) = \frac{G}{\sigma_{xy}(B)}$$

where the large value of G=0.49 signifies a strong coupling between ρ_{xx} and σ_{xy}^{-1}. If electronic conduction with $\mu B \gg 1$ takes place in a single band or N bands, then $\sigma_{xy}(B) = en/B$ or $\sigma_{xy}(B) = \Sigma_i^N en_i/B$, respectively. In the regime where G is field-independent, the magnetoresistance yields:

$$MR = \frac{\rho_{xx}(B) - \rho_{xx}(0)}{\rho_{xx}(0)} \approx GB\mu$$

for conduction in a single band where $\rho_{xx}(0) = 1/en\mu$ or using a population weighted mobility $MR \approx GB\Sigma_i^N \mu_i n_i/n_{total}$ for multiband conduction. The field-independent G captures the linear scaling of the magnetoresistance when varying the magnetic field and mobility both in single- and multiband systems. Using $\mu \approx$ 100,000 cm^2/Vs and $G = 0.49$, we obtain a lower value of $MR(15T) \approx GB\mu = 7350\%$ compared to experiments, which suggests that an additional component influences σ_{xy}(B). The conductivity tensor elements are displayed and discussed further in Supplementary Section 11, where it is shown that σ_{xy} exhibits an inversely proportional field dependence at high magnetic fields, but that it deviates significantly from the conventional conductivity tensor (see Figure S15). As discussed in this supplementary section, the anomalous Hall effect may be a likely candidate for this additional source.

Physical picture of the linear magnetoresistance:
The field-independent G is predicted by the guiding center model (*14*). For itinerant charge carriers strictly confined to a 2D sheet, the semiclassical transport with $\mu_0 B > 1$ and $\xi \gg r_c$ causes the carriers to undergo rapid cyclotron motion while following an overall guiding center motion along the equipotential contours of the disorder potential (Figure S12a). In strict 2D systems, the confinement in the *x*/*y*-plane causes the guiding center motion to follow closed orbits. Relaxing the strict 2D confinement results in an interesting case where an overall motion in the *x*/*y*-plane is enabled by motion in the *z*-direction, which allows carriers to escape the closed orbits by moving across layers with different disorder landscapes (Figure S12b). If the kinetic energy along the *z*-direction only allows movement across layers at locations where the variations in the disorder landscape does not exceed the kinetic energy, the current gets squeezed along *z*. This temporary constraint in the *x*/*y*-plane is associated with a slow guiding center velocity, which leads to the linear magnetoresistance (*14*).
In contrast, if the out-of-plane kinetic energy exceeds the disorder potential variations, free movement across layers

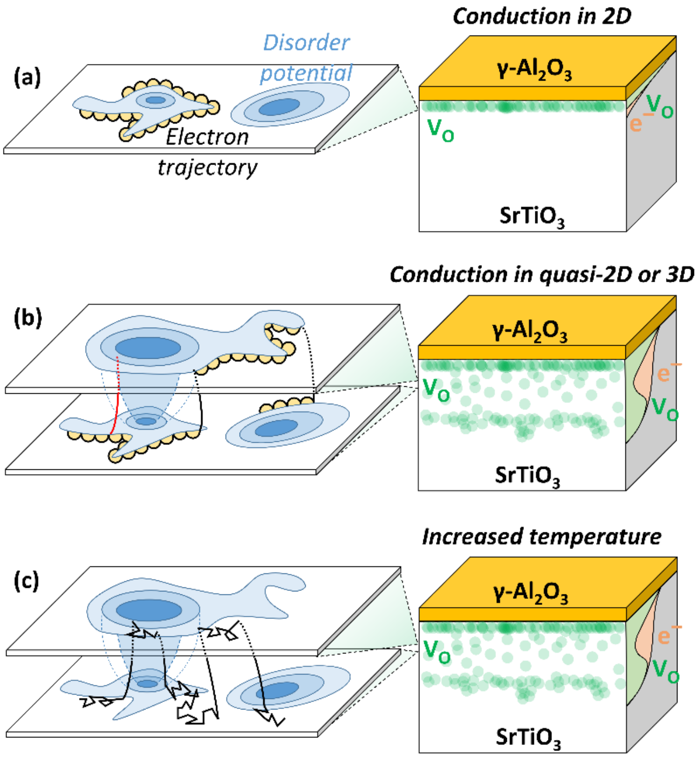

***Figure S12**: Schematics of the proposed mechanism. Schematic illustrations of the semiclassical magnetotransport in high perpendicular magnetic fields when high-mobility electrons (a) are strictly confined in 2D, (b) are restricted to move vertically by a disorder potential in some locations (red vertical line) but allowed in other locations (black vertical lines) and (c) are heated to reduce the mean-free path with the thermal energy exceeding the disorder potential variations, which leads to unrestricted vertical movement.*

is observed, resulting in a magnetoresistance departing from linearity (Figure S12c). This situation arises when the thermal energy of the system increases, which is consistent with the thermal relaxation of the high-resistive state observed in Figure 2b. An additional effect of the increased temperature is enhanced scattering, which may also cause departure from the semiclassical transport regime and the associated linear magnetoresistance.

Defect engineering:

Next, we can consider three ways of tuning the magnetoresistance through defect engineering, namely by using different growth conditions, post-growth annealing in oxygen and sample aging (Figure 4b-c).

(I) Varying the growth conditions:

The conductivity in the γ-Al_2O_3/$SrTiO_3$ heterostructures results from oxygen vacancies formed during the deposition of γ-Al_2O_3 on $SrTiO_3$. By controlling the oxygen partial pressure during the pulsed laser deposition of γ-Al_2O_3 on $SrTiO_3$, the amount of oxygen vacancy defects in $SrTiO_3$ can be controlled, which directly links to the resulting sheet carrier density. Therefore, Figure 4b-c effectively constitutes a defect-property relationship chart where the growth-induced defects determine the resulting mobility and magnetoresistance.

Several studies confirm that the conductivity in the γ-Al_2O_3/$SrTiO_3$ heterostructures originates from oxygen vacancies, including in-situ transport measurements in oxygen or oxygen-poor atmospheres during the γ-Al_2O_3 growth (*20*, *21*), post-growth annealing at various temperatures and environments (*22*, *23*), high-temperature equilibrium conduction measurements (*24*), and angle-resolved photoemission spectroscopy (*15*).

(II) Sample aging:

A boost in the electron mobility at the γ-Al_2O_3/$SrTiO_3$ heterostructure after sample aging at room temperature was previously studied numerically and experimentally (*2*, *10*). Numerically, the oxygen vacancy donors were found to localize at the γ-Al_2O_3/$SrTiO_3$ heterointerface due to broken lattice symmetry. In addition, an oxygen vacancy front was found to gradually diffusive deeper into the bulk of $SrTiO_3$ (*10*) as schematically illustrated by the oxygen vacancy profile in the right panel of Figure S12. For convenience, the simulated results from Ref. (*10*) are shown in Figure S13. The resulting electrostatic potential from the positively charged oxygen vacancies caused the electrons to also shift gradually deeper into $SrTiO_3$ while being primarily located in the region between the interface and the diffusion front (Figure S13a). This sub-interface region formed an 'electron highway' comprising a high concentration of electrons and a low concentration of oxygen vacancy scattering sites after several months of room temperature storage. The self-enhancing high mobility resulting from this dynamic donor-electron separation (Figure S13b) serves to further promote the magnetoresistance through the positive correlation between the mobility and magnetoresistance (Figure 4f). In addition, the aging is also predicted to widen the depth distribution of the electron gas, effectively increasing the out-of-plane motion of the electrons that is a prerequisites for the guiding center model (*14*).

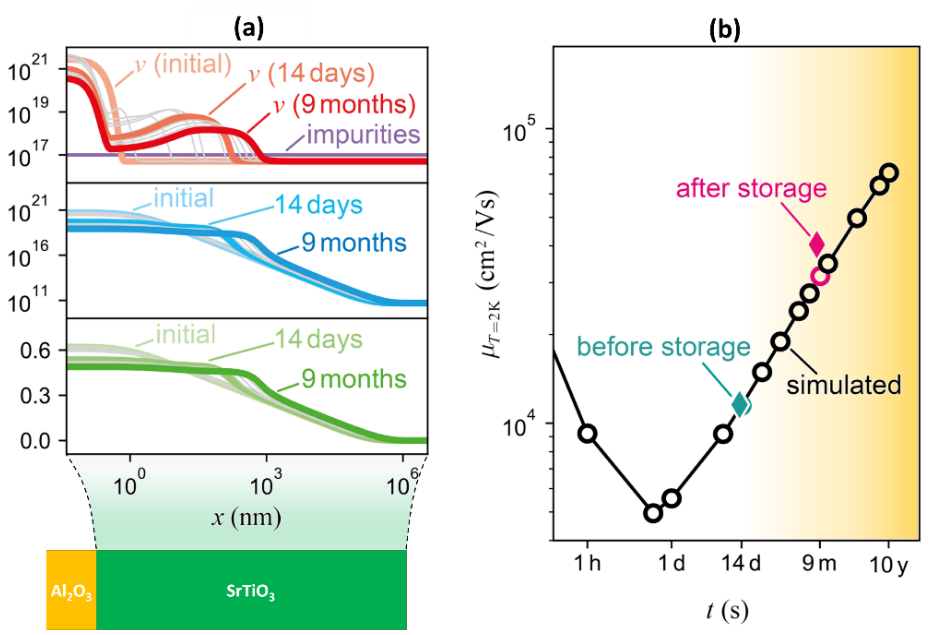

Figure S13: (a) Simulated depth profile of oxygen vacancies (top panel), itinerant electrons (middle panel) and electrostatic potential (lower panel) in γ-Al_2O_3/$SrTiO_3$ as a function of aging time at room temperature. Here, *x* denotes the distance from the interface. **(b)** Electron mobility at 2 K ($\mu_{T=2K}$) as a function of room temperature aging time (*t*). The black markers correspond to simulations whereas the colored markers are experimental values. The sample shown here is different from that presented in Figure 3 and Figure S5, although both are γ-Al_2O_3/$SrTiO_3$ deposited in the same PLD chamber by the same person. The figure is adapted from Ref. (*10*) and reproduced with the present aesthetic modifications performed in Ref. (*25*).

(III) Post-growth annealing:
A detrimental effect is observed after annealing at 200 °C in oxygen where the magnetoresistance is strongly reduced (Figure 3b). We attribute this decrease in magnetoresistance to a lowering of the electron mobility (Figure S8) which concurs with an increase in the effective disorder as justified below.
In a recent study, scanning SQUID measurements conducted on disordered $LaAlO_3/SrTiO_3$ heterostructures were used to track the low-temperature current distribution after consecutive post-growth annealing steps in oxygen (*26*), similar to those applied in Figure 3b. As the conductivity originates from thermodynamically unstable oxygen vacancies in both γ-$Al_2O_3/SrTiO_3$ and disordered $LaAlO_3/SrTiO_3$ (*22*, *24*), the annealing reduced the itinerant carrier density in both material systems by annihilation of oxygen vacancies. Across the consecutive annealing steps, the current distribution measured using the scanning SQUID measurements transitioned from weakly modulated around current holes to heavily modulated around both previous and newly generated current holes (*26*). Eventually, the current showed a strongly disordered filament-like current flow close to the metal/insulator transition induced by the annealing process. This behavior was attributed primarily to a lowering of the Fermi energy level with respect to the disorder potential (*26*). Interestingly, according to the guiding center model, the Hall angle in 3D Dirac metals can be tuned by varying the Fermi energy level with respect to the characteristic disorder potential variations as $\tan(\theta) \propto (E_f/eV_0)^{3/2}$ (*14*). By comparison with our present result (Figure S8d), we find that $|\tan(\theta)|$ drops by a factor of 2 in γ-$Al_2O_3/SrTiO_3$ upon annealing at conditions found to lower the Fermi energy level with respect to the disorder potential disordered $LaAlO_3/SrTiO_3$. This establishes annealing and the associated defect engineering as a knob to dynamically control the effective degree of disorder and the XMR performance. By comparison with the scanning SQUID study, the evolution during the annealing also suggests that the disorder does not arise from isolated and thermodynamically unstable oxygen vacancies but may rather be caused by oxygen vacancy clusters or other extended defects. This is consistent with the large characteristic length of the disorder potential.

Supplementary Section 11: Geometric dependence of the magnetoresistance

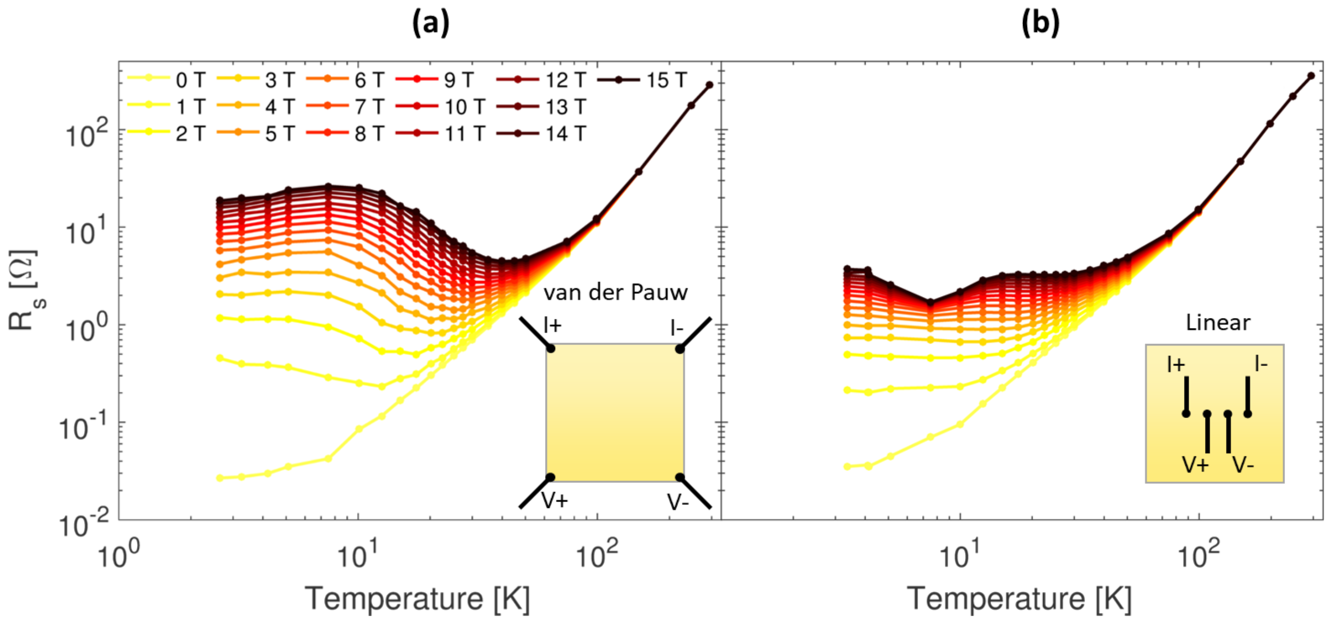

Figure S14: *Sheet resistance (R_s) as a function of temperature and magnetic fields in* ***(a)*** *a standard van der Pauw geometry of a 5 mm x 5 mm sample and* ***(b)*** *a linear bond configuration in the center of the same sample. The temperature dependence of the zero-field sheet resistance is consistent across the two measurement configurations. When a magnetic field is applied, a large magnetoresistance is observed in both cases at low temperatures, but significant differences in the behavior is observed for T < 40 K resulting from the geometric contribution to the magnetoresistance.*

Supplementary Section 12: Field-dependence of the conductivity tensor elements

The conductivity tensor components can be calculated from the measured resistances by inverting the resistivity tensor:

$$\sigma_{xy} = \frac{\rho_{xy}}{\rho_{xy}^2 + \rho_{xx}^2}$$

$$\sigma_{xx} = \frac{\rho_{xx}}{\rho_{xy}^2 + \rho_{xx}^2}$$

The conductivity tensor elements normalized with the electron gas thickness t are shown in Figure S15a-b. The results may be compared to the conductivity tensor for a single-band electronic system in the relaxation time approximation:

$$\bar{\bar{\sigma}} = \frac{en\mu}{1+(\mu B)^2}\begin{bmatrix} 1 & -\mu B \\ \mu B & 1 \end{bmatrix} \quad (1)$$

The transverse and longitudinal components are plotted in Figure S15c-d. For $\mu B \ll 1$, we obtain

$$|\sigma_{xy}| \sim en\mu^2 B \propto B^1$$

$$\sigma_{xx} \sim en\mu \propto B^0$$

with the proportional relationships consistent with those observed at high temperatures in Figure S15a-b. For $\mu B \gg 1$, we obtain

$$|\sigma_{xy}| \sim \frac{en}{B} \propto B^{-1}$$

$$\sigma_{xx} \sim \frac{en}{\mu B^2} \propto B^{-2}$$

Interestingly, the experimental σ_{xy} follows this trend for very high values of μB obtained at low temperatures and high magnetic fields, but a large range of intermediate field values yields a $B^{-1.5}$ scaling behavior suggesting that an additional contribution is at play. In addition, the high-field behavior of σ_{xx} also deviates from Equation (1). The deviations are consistent with the lack of magnetoresistance if transport is solely described by Equation (1), as

$$\rho_{xx}(B) = \frac{\sigma_{xx}}{\sigma_{xy}^2 + \sigma_{xx}^2} = \frac{\frac{en\mu}{1+(\mu B)^2}}{\left(\frac{en\mu}{1+(\mu B)^2}\right)^2 \mu^2 B^2 + \left(\frac{en\mu}{1+(\mu B)^2}\right)^2} = \frac{1+(\mu B)^2}{(1+(\mu B)^2)en\mu} = \frac{1}{en\mu}$$

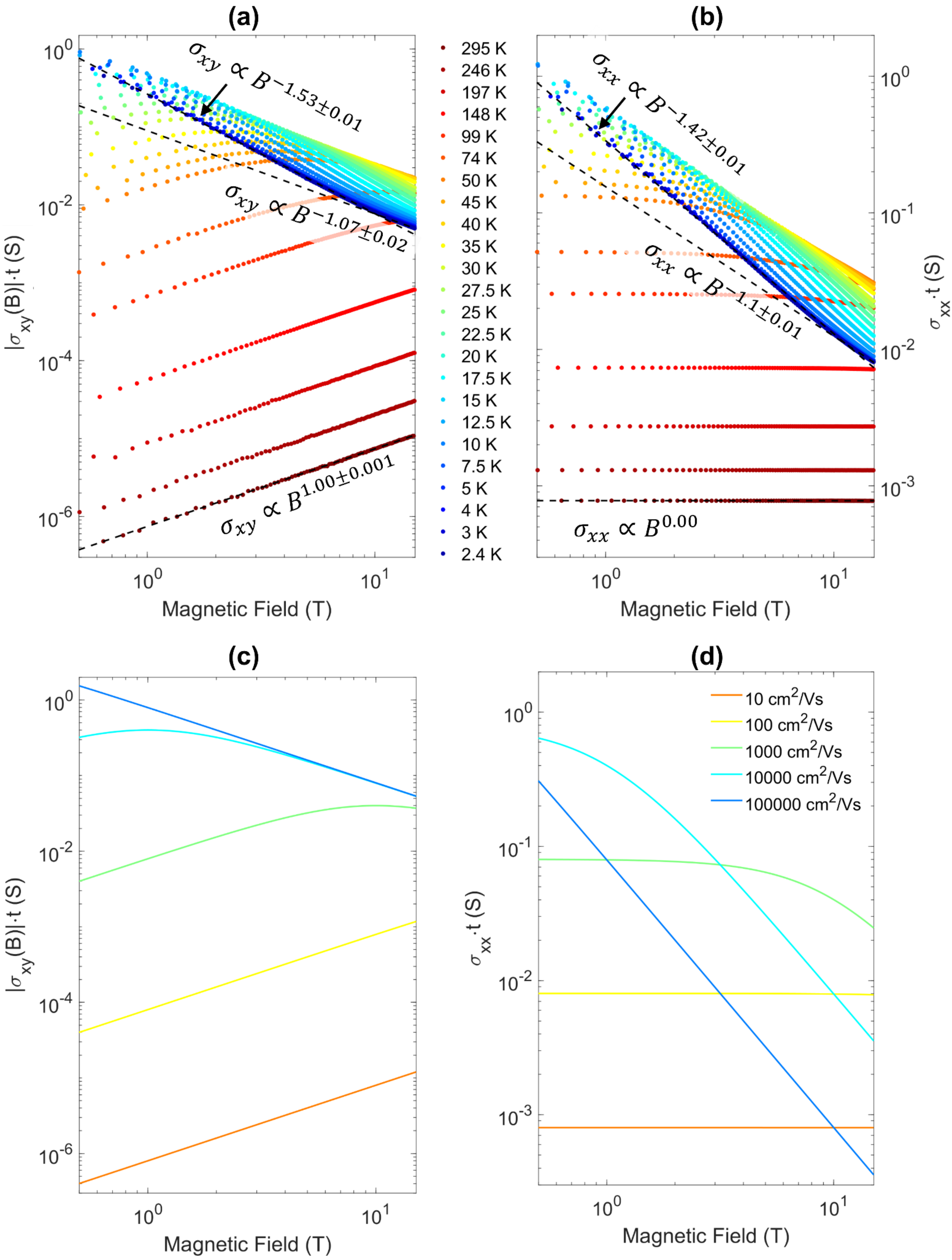

***Figure S15: (a)** Transverse and **(b)** longitudinal conductivity tensor elements (multiplied by the thickness of the electron gas t) as a function of magnetic field for various temperatures for the same γ-Al_2O_3/$SrTiO_3$ sample as displayed in the main text. The conductivity tensor elements are obtained by inverting the measured resistivity tensor element. **(c)** Transverse and **(d)** longitudinal conductivity elements extracted for the single-band conductivity tensor (Equation 1) as a function of the magnetic field for various electron mobilities using a sheet carrier density of* $5 \cdot 10^{14}\ cm^{-2}$.

If the factor G is independent of the magnetic field, the longitudinal resistance follows that of the transverse conductivity:

$$\rho_{xx}(B) = \frac{G}{\sigma_{xy}(B)}$$

Using this expression and $\rho_{xx}(B=0) = 1/\sigma_{xx}(B=0)$, the magnetoresistance can be extracted for the general case of multiple bands, given that there are no additional physical phenomena such as an anomalous Hall effect:

$$MR = \frac{\rho_{xx}(B) - \rho_{xx}(0)}{\rho_{xx}(0)} = \frac{G\sigma_{xx}(0)}{\sigma_{xy}} - 1 = \frac{G\sum_i en_i\mu_i}{\sum_i \frac{en_i\mu_i^2 B}{(1+\mu_i B)^2}} - 1 \overset{\mu_i B \gg 1}{\sim} GB\frac{\sum_i n_i\mu_i}{\sum_i n_i} \approx GB\sum_i \mu_i \frac{n_i}{n_{total}}$$

where the sum is taken over all the bands. In the single-band case, it reduces to:

$$MR \approx GB\mu$$

which reproduces the results of Ref. (*14*). Using $\mu \approx 100{,}000$ cm^2/Vs and $G = 0.49$, as relevant for γ-Al_2O_3/$SrTiO_3$, we obtain a value of $MR(15T) \approx GB\mu = 7350\%$, which is roughly an order of magnitude lower than the experimental value. As G and B are experimentally determined values with a bound of $G \leq 0.5$, this discrepancy can only be explained by either one or several of the bands having much higher mobility or that a key assumption in the derivation is violated. A mobility on the order of 1,000,000 cm^2/Vs in $SrTiO_3$ is deemed unrealistic, both considering the present magnetotransport data as well as the bulk literature data on mobility in $SrTiO_3$ (*11*, *27*). Therefore, we attribute the discrepancy to a key assumption being violated. The comparison between Figure S15a and c reveals that the experimental transverse conductivity at *B* = 15 T is around an order of magnitude smaller than that of Equation (1). This deviation of the transverse conductivity may be caused by the anomalous Hall effect. For the sample exhibiting the highest magnetoresistance, we observe stripy magnetic ordering emerging at the same temperature as the non-linear Hall coefficient (*4*). Above this critical temperature, the Hall coefficient is linearly dependent on the magnetic field. As the sample is cooled down, the weak-field Hall coefficient retained the same linear dependence, but at high fields the Hall coefficient was drastically strengthened. Such behavior has previously been attributed to the anomalous Hall effect, which leads to an increase in the Hall coefficient compared to the linear weak-field response (*4*) and could be the origin of the additional source entering σ_{xy}(B).

Supplementary Section 13: Anisotropy

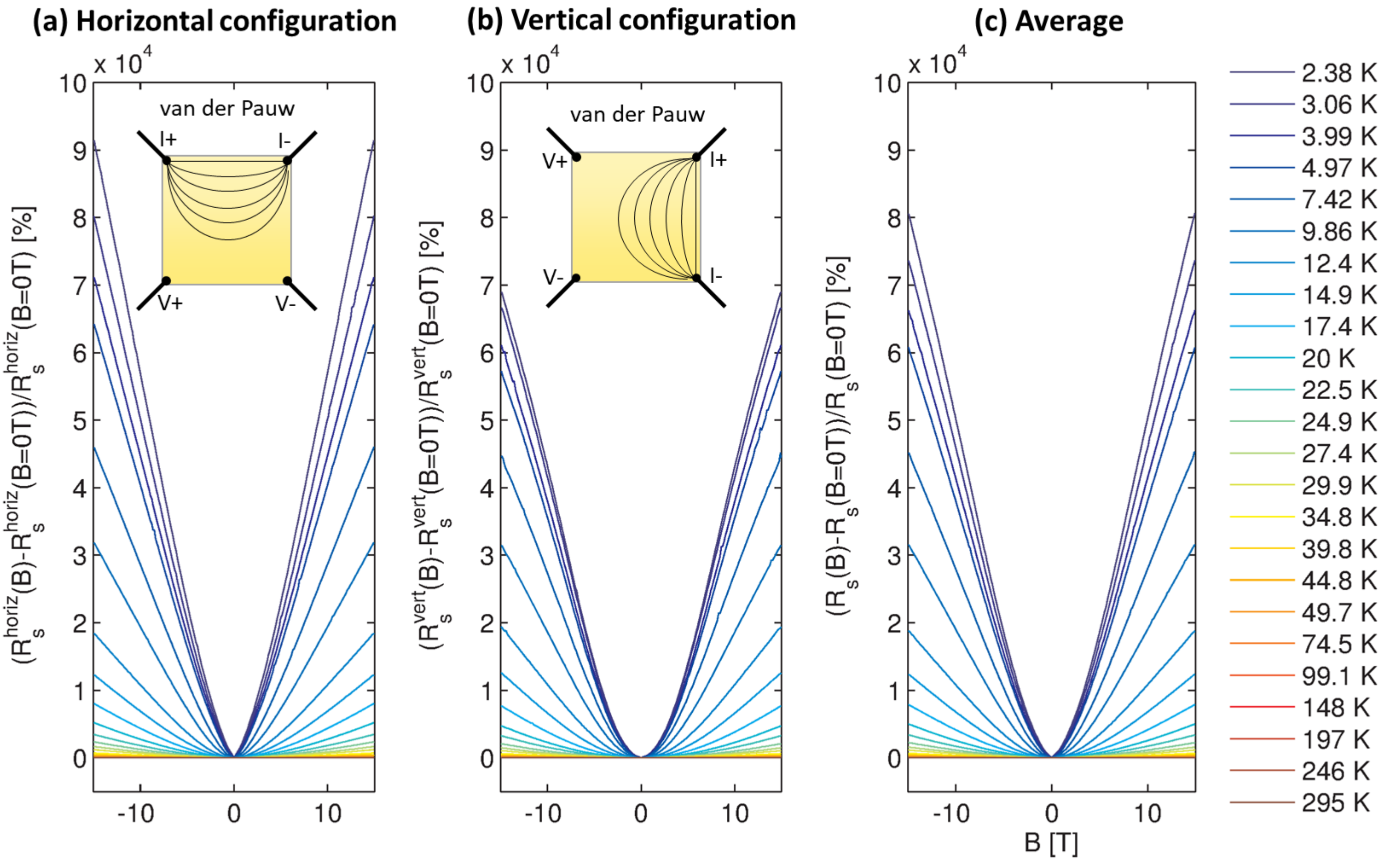

Figure S16: *Magnetoresistance measured in van der Pauw geometry with* ***(a)*** *horizontal and* ***(b)*** *vertical contact configurations as well as the averaged magnetoresistance. An anisotropy on the order of ±10% is observed in the magnetoresistance measured at temperatures below approximately 7 K whereas higher temperatures result in less anisotropy.*

Supplementary Section 14: References

1. F. Fallah Tafti, Q. Gibson, S. Kushwaha, J. W. Krizan, N. Haldolaarachchige, R. J. Cava, Temperature−field phase diagram of extreme magnetoresistance. *Proc. Natl. Acad. Sci.* **113** (2016).

2. P. Schütz, D. V. Christensen, V. Borisov, F. Pfaff, P. Scheiderer, L. Dudy, M. Zapf, J. Gabel, Y. Z. Chen, N. Pryds, V. A. Rogalev, V. N. Strocov, T.-L. Lee, H. O. Jeschke, R. Valentí, M. Sing, R. Claessen, Microscopic origin of the mobility enhancement at a spinel/perovskite oxide heterointerface revealed by photoemission spectroscopy. *Phys. Rev. B* **96**, 161409 (2017).

3. D. V. Christensen, Y. Frenkel, P. Schütz, F. Trier, S. Wissberg, R. Claessen, B. Kalisky, A. Smith, Y. Z. Chen, N. Pryds, Electron Mobility in γ-$Al_2O_3/SrTiO_3$. *Phys. Rev. Appl.* **9**, 054004 (2018).

4. D. V. Christensen, Y. Frenkel, Y. Z. Chen, Y. W. Xie, Z. Y. Chen, Y. Hikita, A. Smith, L. Klein, H. Y. Hwang, N. Pryds, B. Kalisky, Strain-tunable magnetism at oxide domain walls. *Nat. Phys.* **15**, 269–274 (2019).

5. R. Niu, W. K. Zhu, Materials and possible mechanisms of extremely large magnetoresistance: a review. *J. Phys. Condens. Matter* **34**, 113001 (2022).

6. R. Lou, Y. F. Xu, L.-X. Zhao, Z.-Q. Han, P.-J. Guo, M. Li, J.-C. Wang, B.-B. Fu, Z.-H. Liu, Y.-B. Huang, P. Richard, T. Qian, K. Liu, G.-F. Chen, H. M. Weng, H. Ding, S.-C. Wang, Observation of open-orbit Fermi surface topology in the extremely large magnetoresistance semimetal $MoAs_2$. *Phys. Rev. B* **96**, 241106 (2017).

7. A. A. Abrikosov, Galvanomagnetic phenomena in metals in the quantum limit. *Sov. Phys. JETP* **29**, 746 (1969).

8. A. A. Abrikosov, Quantum magnetoresistance. *Phys. Rev. B* **58**, 2788–2794 (1998).

9. J. Yue, Y. Ayino, T. K. Truttmann, M. N. Gastiasoro, E. Persky, A. Khanukov, D. Lee, L. R. Thoutam, B. Kalisky, R. M. Fernandes, V. S. Pribiag, B. Jalan, Anomalous transport in high-mobility superconducting $SrTiO_3$ thin films. *Sci. Adv.* **8**, eabl5668 (2022).

10. A. F. Zurhelle, D. V. Christensen, S. Menzel, F. Gunkel, Dynamics of the spatial separation of electrons and mobile oxygen vacancies in oxide heterostructures. *Phys. Rev. Mater.* **4**, 104604 (2020).

11. F. Trier, D. V. Christensen, N. Pryds, Electron mobility in oxide heterostructures. *J. Phys. Appl. Phys.* **51**, 293002 (2018).

12. M. M. Parish, P. B. Littlewood, Non-saturating magnetoresistance in heavily disordered semiconductors. *Nature* **426**, 162–165 (2003).

13. M. M. Parish, P. B. Littlewood, Classical magnetotransport of inhomogeneous conductors. *Phys. Rev. B* **72**, 094417 (2005).

14. J. C. W. Song, G. Refael, P. A. Lee, Linear magnetoresistance in metals: Guiding center diffusion in a smooth random potential. *Phys. Rev. B* **92**, 180204 (2015).

15. A. Chikina, D. V. Christensen, V. Borisov, M.-A. Husanu, Y. Chen, X. Wang, T. Schmitt, M. Radovic, N. Nagaosa, A. S. Mishchenko, R. Valentí, N. Pryds, V. N. Strocov, Band-Order Anomaly at the γ-$Al_2O_3/SrTiO_3$ Interface Drives the Electron-Mobility Boost. *ACS Nano* **15**, 4347–4356 (2021).

16. Y. Z. Chen, N. Bovet, F. Trier, D. V. Christensen, F. M. Qu, N. H. Andersen, T. Kasama, W. Zhang, R. Giraud, J. Dufouleur, T. S. Jespersen, J. R. Sun, A. Smith, J. Nygård, L. Lu, B. Büchner, B. G. Shen, S. Linderoth, N. Pryds, A high-mobility two-dimensional electron gas at the spinel/perovskite interface of γ-$Al_2O_3/SrTiO_3$. *Nat. Commun.* **4**, 1371 (2013).

17. P. Schütz, F. Pfaff, P. Scheiderer, Y. Z. Chen, N. Pryds, M. Gorgoi, M. Sing, R. Claessen, Band bending and alignment at the spinel/perovskite γ–Al_2O_3/$SrTiO_3$ heterointerface. *Phys. Rev. B* **91**, 165118 (2015).

18. M. Yazdi-Rizi, P. Marsik, B. P. P. Mallett, A. Dubroka, D. V. Christensen, Y. Z. Chen, N. Pryds, C. Bernhard, Infrared ellipsometry study of the confined electrons in a high-mobility γ-Al_2O_3/$SrTiO_3$ heterostructure. *EPL Europhys. Lett.* **113**, 47005 (2016).

19. E. Sawaguchi, A. Kikuchi, Y. Kodera, Dielectric Constant of Strontium Titanate at Low Temperatures. *J. Phys. Soc. Jpn.* **17**, 1666 (1962).

20. M. von Soosten, Dennis. V. Christensen, C.-B. Eom, Thomas. S. Jespersen, Y. Chen, N. Pryds, On the emergence of conductivity at $SrTiO_3$-based oxide interfaces – an in-situ study. *Sci. Rep.* **9**, 18005 (2019).

21. T. Hvid-Olsen, C. Gadea, F. B. Holde, K. M. Hoffmann, T. S. Jespersen, K. Grove-Rasmussen, F. Trier, D. V. Christensen, Spatial control of the conductivity in $SrTiO_3$ -based heterointerfaces using inkjet printing. *J. Phys. Energy* **4**, 044005 (2022).

22. D. V. Christensen, M. von Soosten, F. Trier, T. S. Jespersen, A. Smith, Y. Chen, N. Pryds, Controlling the carrier density of $SrTiO_3$-based heterostructures with annealing. *Adv. Electron. Mater.* **3**, 1700026 (2017).

23. T. Steegemans, S. Yun, C. N. Lobato, E. Brand, Y. Chen, F. Trier, D. V. Christensen, Tuning Oxide Properties by Oxygen Vacancy Control During Growth and Annealing. *J. Vis. Exp.*, 58737 (2023).

24. F. Gunkel, S. Hoffmann-Eifert, R. A. Heinen, D. V. Christensen, Y. Z. Chen, N. Pryds, R. Waser, R. Dittmann, Thermodynamic Ground States of Complex Oxide Heterointerfaces. *ACS Appl. Mater. Interfaces* **9**, 1086–1092 (2017).

25. D. V. Christensen, Perspectives on oxide heterostructures – the curious case of γ-Al_2O_3/$SrTiO_3$. *Nanoscale* **15**, 3704 (2023).

26. A. V. Bjørlig, D. V. Christensen, R. Erlandsen, N. Pryds, B. Kalisky, Current Mapping of Amorphous $LaAlO_3$/$SrTiO_3$ near the Metal–Insulator Transition. *ACS Appl. Electron. Mater.* **4**, 3421–3427 (2022).

27. F. Trier, K. V. Reich, D. V. Christensen, Y. Zhang, H. L. Tuller, Y. Chen, B. I. Shklovskii, N. Pryds, Universality of electron mobility in $LaAlO_3$/$SrTiO_3$ and bulk $SrTiO_3$. *Appl. Phys. Lett.* **111**, 092106 (2017).
